# Lithium induces mesenchymal-epithelial differentiation during human kidney development by activation of the Wnt signalling system

**DOI:** 10.1038/s41420-017-0021-6

**Published:** 2018-02-07

**Authors:** Karen L. Price, Maria Kolatsi-Joannou, Chiara Mari, David A. Long, Paul J. D. Winyard

**Affiliations:** Developmental Biology and Cancer Programme, UCL Great Ormond Street Institute of Child Health, London, UK

## Abstract

Kidney function is directly linked to the number of nephrons which are generated until 32–36 weeks gestation in humans. Failure to make nephrons during development leads to congenital renal malformations, whilst nephron loss in adulthood occurs in progressive renal disease. Therefore, an understanding of the molecular processes which underlie human nephron development may help design new treatments for renal disease. Mesenchyme to epithelial transition (MET) is critical for forming nephrons, and molecular pathways which control rodent MET have been identified. However, we do not know whether they are relevant in human kidney development. In this study, we isolated mesenchymal cell lines derived from human first trimester kidneys in monolayer culture and investigated their differentiation potential. We found that the mesenchymal cells could convert into osteogenic, but not adipogenic or endothelial lineages. Furthermore, addition of lithium chloride led to MET which was accompanied by increases in epithelial (*CDH1*) and tubular (*ENPEP*) markers and downregulation of renal progenitor (*SIX2*, *EYA1*, *CD133*) and mesenchymal markers (*HGF*, *CD24*). Prior to phenotypic changes, lithium chloride altered Wnt signalling with elevations in *AXIN2*, GSK3β phosphorylation and β-catenin. Collectively, these studies provide the first evidence that lithium-induced Wnt activation causes MET in human kidneys. Therapies targeting Wnts may be critical in the quest to regenerate nephrons for human renal diseases.

## Introduction

Healthy mature human kidneys contain around 1 million nephrons^1, 2^, yet they originate from only a few hundred cells during development. The human metanephros initiates at 5 weeks of gestation when the epithelia of the ureteric bud invades the metanephric mesenchyme^3^. Subsequently, the ureteric bud branches multiple times to generate the collecting duct system, whereas the mesenchyme condenses into aggregates that epithelialise to form podocytes, proximal tubules and loops of Henle^3^. Therefore, a critical early step in nephron formation is mesenchymal-epithelial transformation (MET)^4^ and defects in this process give rise to congenital anomalies such as dysplastic kidneys, which contain reduced numbers of functioning nephrons and other maldevelopments such as metaplasia^5^. When severe, dysplastic malformations cause childhood chronic kidney disease (CKD), which can only be treated via dialysis or kidney transplantation at present. Several studies in rodents and humans have also demonstrated that lower nephron numbers in adulthood increase the susceptibility of an individual to develop hypertension, albuminuria and CKD^6–8^. This is a major health problem with CKD now affecting up to 13% of the adult population in the Western world^9^.

Studies in rodents have identified that a critical signalling pathway that can induce MET is the activation of canonical wingless-related integration site (Wnt) signalling leading to glycogen synthase kinase-3β (GSK-3β) inhibition and stabilisation of β-catenin. Exposure of isolated rodent mesenchymes to either of the GSK-3β inhibitors, lithium or 6-bromoindirubin-3′-oxime (BIO), results in epithelial differentiation^10–12^. Furthermore, transgenic mice with a complete loss of *Wnt4*^13^ or lack of β-catenin activity specifically in renal mesenchymal progenitor cells are unable to form mature renal epithelial structures^14^. However, we do not yet know whether the activation of canonical Wnt signalling is relevant in human foetal kidney development. To examine this, we used multiple human renal mesenchymal cell lines and exposed them to lithium chloride. Addition of lithium led to human MET in vitro accompanied by activation of Wnt signalling pathways. These findings suggest that modulating Wnt signalling may be critical in the quest to regenerate nephrons for human CKD.

## Results

### Assessment of the differentiation potential of foetal kidney mesenchymal cells

**Fig. 1:**
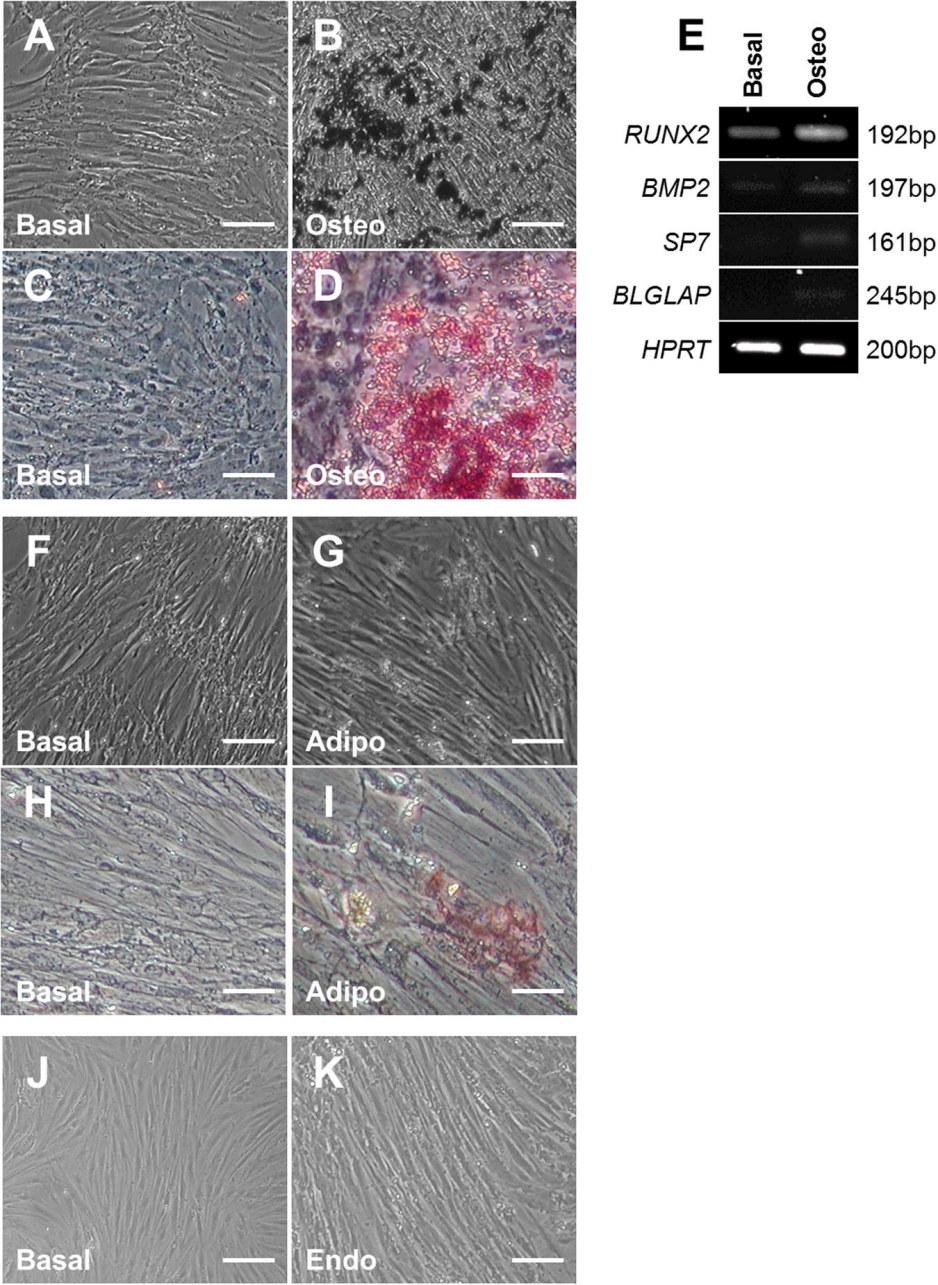
Differentiation potential of human foetal mesenchymal cells. Human foetal kidney mesenchymal cells isolated from 10 week gestation foetuses and grown in basal media for 12 days had an irregular elongated shape (**a**). Mesenchymal cells grown in osteogenic (osteo) media showed extensive calcium deposition (**b**). In contrast, to cells grown in basal media (**c**), those exposed to osteogenic media were strongly positive for Alizarin red (**d**). Examination of mRNA levels (**e**) of runt-related transcription factor 2 (*RUNX2*), bone morphogenetic protein 2 (*BMP2*), osterix (*SP7*) and osteocalcin (*BLGLAP*) in one cell line. Hypoxanthine-guanine phosphoribosyltransferase (*HPRT)* was used as a housekeeping gene. No morphological differences were observed between cells grown in either basal (**f**) or adipogenic (adipo, **g**) media for 21 days; positive lipid-rich vacuoles were also not detected in either condition by Oil Red O staining (**h**) and (**i**). No overt phenotypic changes were detected between cells grown on Matrigel for 7 days in either basal (**j**) or endothelial (endo, **k**) media. Bar is 100 µm in all panels. Images are representative of experiments performed in triplicate on three independent cell lines

Three human metanephric cell lines were generated as previously described^15, 16^ from different 10 week gestation fetuses. Foetal renal mesenchymal cells have the capacity to differentiate into renal epithelia, stroma and endothelia in vivo, but it is uncertain whether they may also be able to generate other lineages. Hence, we initially examined potential differentiation in several pathways here, namely osteogenic, adipogenic and vascular lineages. To evaluate osteogenic differentiation, the three different mesenchymal cell lines were grown either in basal media alone or StemMACS OsteoDiff media. At the start of the observation period, cells all had a typical ‘mesenchymal’ morphology with irregular, elongated outlines^15^. Over the next twelve days the cultures grown in basal media became confluent but we observed no gross changes in cell morphology (Fig. 1a). In contrast, cells from all three lines grown in osteogenic media, appeared to contain extensive calcium deposits (Fig. 1b), which was confirmed by Alizarin red staining^17^ (Fig. 1c, d). We also examined the mRNA expression of genes associated with osteogenic differentiation^18^ namely runt-related transcription factor 2 (*RUNX2)*, bone morphogenetic protein 2 (*BMP2)*, osterix (*SP7)* and osteocalcin (*BLGLAP*) using reverse-transcriptase PCR in one of our cell lines. We found that all these genes were either absent or detected at very low levels in cells exposed to basal media, but increased in osteogenic media (Fig. 1e).

Next, we assessed adipogenic differentiation by exposing the mesenchymal cells lines to StemMACS AdipoDiff media. After twenty-one days of culture, we did not observe any morphological differences between cells growth in either basal (Fig. 1f) or adipogenic media (Fig. 1g). Furthermore, the lipid-rich vacuoles characteristic of adipocytes^19^ were not present in either condition, as assessed with Oil Red O solution (Fig. 1h, i). We also seeded the mesenchymal cells onto matrigel and exposed them to endothelial growth media to promote differentiation into vascular cells. After seven days, however, no overt phenotypic changes were observed between cells grown in either basal or endothelial media (Fig. 1j–k**)**.

### Mesenchyme to epithelial differentiation following exposure to lithium chloride

**Fig. 2:**
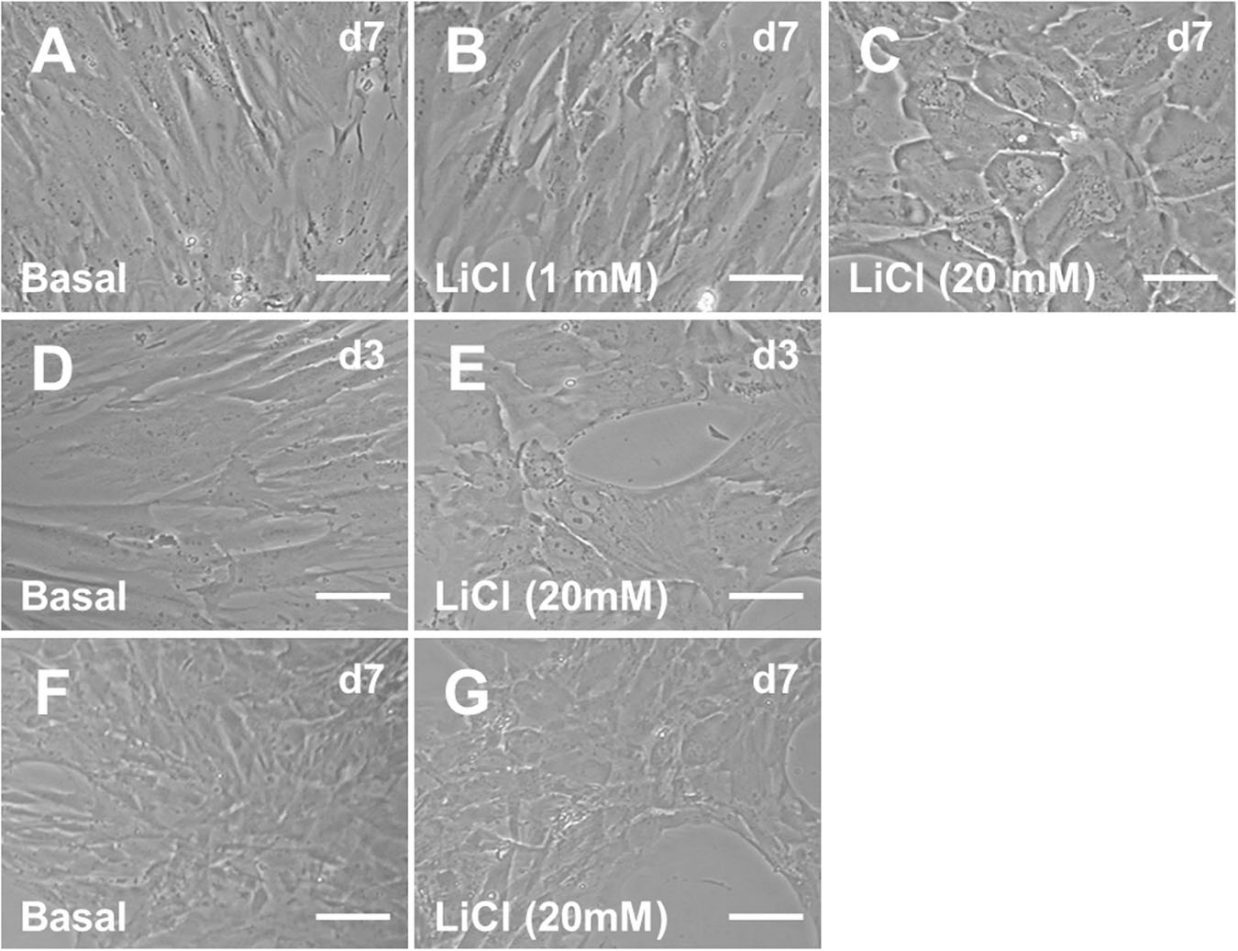
Exposure of foetal kidney mesenchymal cells to lithium chloride. Kidney mesenchymal cells isolated from 10 week gestation foetuses and exposed to basal medium (**a**), and 1 mM lithium chloride (LiCl, **b**) for seven days (d7) had similar morphology. Stimulation with 20 mM of LiCl (**c**) led to the appearance of islands of epithelial-like cells. When cells were exposed to basal medium for three days (d3) they had a mesenchymal appearance (**d**), whilst exposure to 20 mM Lithium reproducibly generated cells with epithelial morphology (**e**) in all three lines. Short-term treatment for only 24 h with basal (**f**) or 20 mM LiCl (**g**) followed by subsequent incubation in basal media for a further six days, resulted in the cells exposed to LiCl having an epithelial morphology by day 7. Bar is 100 µm in all panels. Images are representative of experiments performed in triplicate on three independent cell lines

Next, we assessed whether any of the cell lines had the potential to undergo MET using lithium chloride; which activates Wnt signalling and causes epithelial differentiation in rodent kidneys^10, 11^. Exposure of human foetal mesenchymal cells to 1 mM lithium chloride for seven days did not cause any change in cell morphology compared with cells grown in basal media (Fig. 2a, b). In contrast, in cells stimulated with 20 mM of lithium chloride for seven days we consistently observed islands of epithelial-like cells (Fig. 2c). To test if epithelisation might be driven by non-lithium aspects of the intervention (e.g.,) alterations in osmotic environment, we also examined the effect of potassium chloride (20 mM) on cell differentiation. In contrast to lithium, addition of potassium chloride had no effect on cell morphology (Supplementary Fig. 1).

We then examined the time-course of mesenchyme to epithelial differentiation following exposure to 20 mM lithium chloride in more detail; cells with epithelial morphology were detected as early as three days after stimulation (Fig. 2d, e). Strikingly, epithelial colonies were observed at day 7 even after a limited exposure to 20 mM lithium chloride for only the first twenty-four hours of culture, followed by a switch to basal medium (Fig. 2f–g), raising the possibility that lithium chloride may be more important in early initiation of MET than in maintaining differentiation.

### Effect of lithium chloride on foetal kidney mesenchymal cell gene expression

**Fig. 3:**
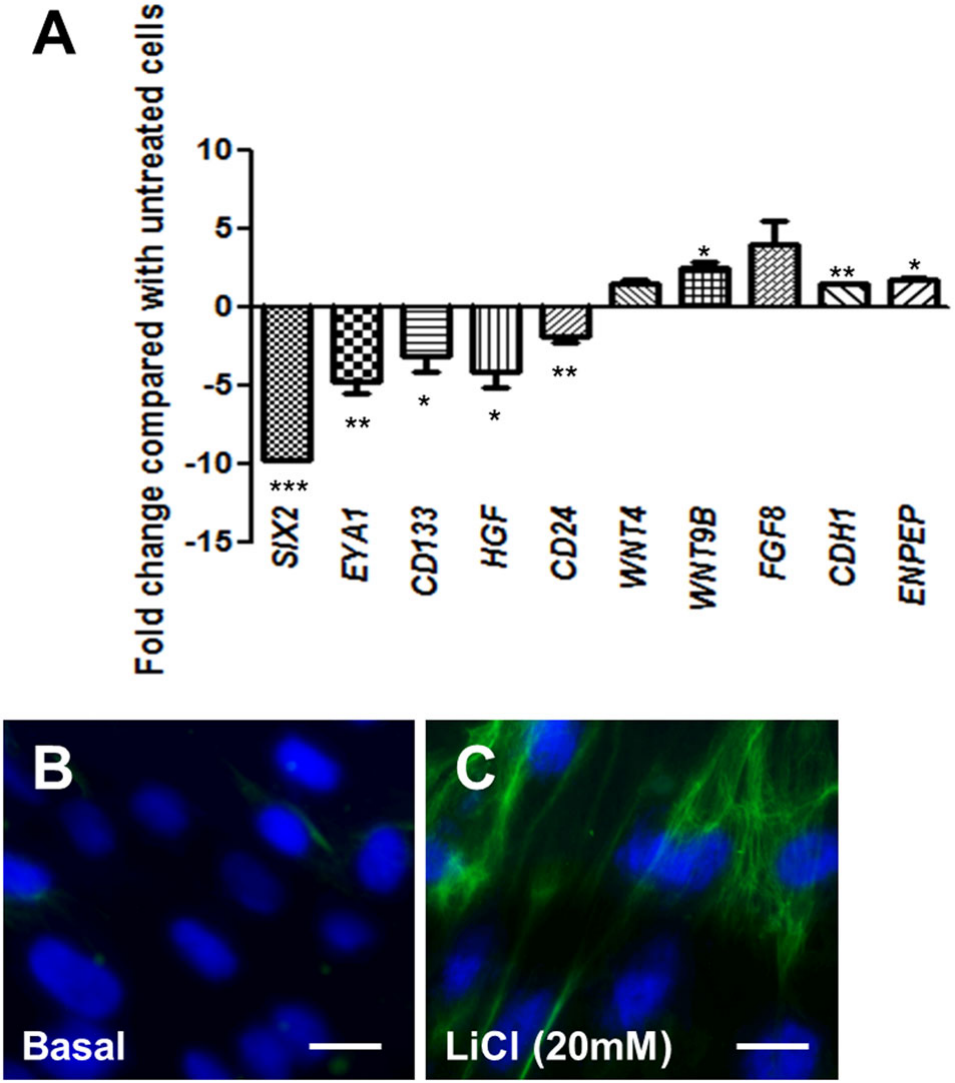
Molecular changes in foetal kidney mesenchymal cells exposed to lithium chloride. **a** qRT-PCR to assess the effect of 7 days of lithium chloride exposure on the mRNA levels of sine oculis homeobox homologue 2 (*SIX2)*, eyes absent homologue 1 (*EYA1)*, prominin-1 (*CD133)*, hepatocyte growth factor (*HGF)*, cluster of differentiation 24 (*CD24)*, wingless-related integration site 4 (*WNT4)*, *WNT9B*, fibroblast growth factor 8 (*FGF8)*, E-cadherin (*CDH1)* and aminopeptidase A (*ENPEP)* in foetal kidney mesenchymal cells. Data is expressed as mean ± SEM of the fold-change in mRNA expression in the lithium chloride treated cells relative to the mean intensity in the three independent untreated metanephric cell lines which were given a standardised value of 1. All measurements were performed in duplicate for three replicates of each cell line. *p < 0.05, **p < 0.01 and ***p < 0.001 between lithium-treated and control-treated cells. Immunocytochemistry for cytokeratin in untreated (**b**) and lithium chloride exposed (**c**) foetal kidney mesenchyme cells. Images are representative of experiments performed on three independent cell lines. Bar is 50 µm in panels **b** and **c**

Next, we examined changes in gene expression using three replicates in each of the three independent mesenchymal cell lines following stimulation with 20 mM lithium chloride for seven days by qRT-PCR (Fig. 3a). mRNA levels of the renal progenitor markers sine oculis homeobox homologue 2 (*SIX2)*^20^ (9.7 ± 0.1-fold, *p* < 0.001), eyes absent homologue 1 (*EYA1)*^20^ (4.8 ± 0.7-fold, *p* < 0.01) and prominin-1 (*CD133)*^21^ (3.1 ± 1.0-fold, *p* < 0.05) were significantly decreased in cells exposed to lithium chloride compared with untreated cells. Mesenchymal markers, hepatocyte growth factor (*HGF)*^22^ (4.1 ± 1.0-fold, *p* < 0.05) and cluster of differentiation 24 (*CD24)*^23^ (1.9 ± 0.4-fold, *p* < 0.01) were also significantly reduced in lithium chloride-treated cells. Furthermore, we assessed secretion of HGF protein into the media of foetal mesenchymal cells with or without exogenous lithium chloride stimulation for seven days, using ELISA. In accord with our qRT-PCR observations, we found a 6.0 ± 1.5-fold decrease in HGF secretion following lithium chloride exposure (*p* < 0.01). We then explored the transcript levels of growth factors implicated in nephron differentiation and found that *WNT9B*^24^ was significantly increased by 2.4 ± 0.5-fold (*p* < 0.05) in cells exposed to lithium chloride. Two other nephrogenic growth factors, fibroblast growth factor 8 (*FGF8)*^25^ (4.0 ± 1.4-fold) and *WNT4*^13^ (1.4 ± 0.3-fold) were also elevated in cells treated with lithium chloride, but neither of these observations were significant compared with untreated cells (*p* = 0.10 and 0.22, respectively). Finally, we assessed the expression of markers of epithelial differentiation and found a modest but significant increase in both E-cadherin (*CDH1)* (1.5 ± 0.1-fold, *p* < 0.01) and the proximal tubule marker, aminopeptidase A (*ENPEP)*^26^ (1.7 ± 0.2-fold, *p* < 0.05). Furthermore, we observed induction of cytokeratin expression by immunocytochemistry in cells exposed to lithium chloride (Fig. 3b–c). We could not detect transcripts for nephrin (*NPHS1*) or podocin (*NPHS2*), markers of podocytes, the specialised epithelial cells of the glomerular filtration barrier^27^, by qRT-PCR in foetal mesenchymal cells with or without lithium chloride treatment.

### Localisation of SIX2 and ENPEP in developing human metanephroi

**Fig. 4:**
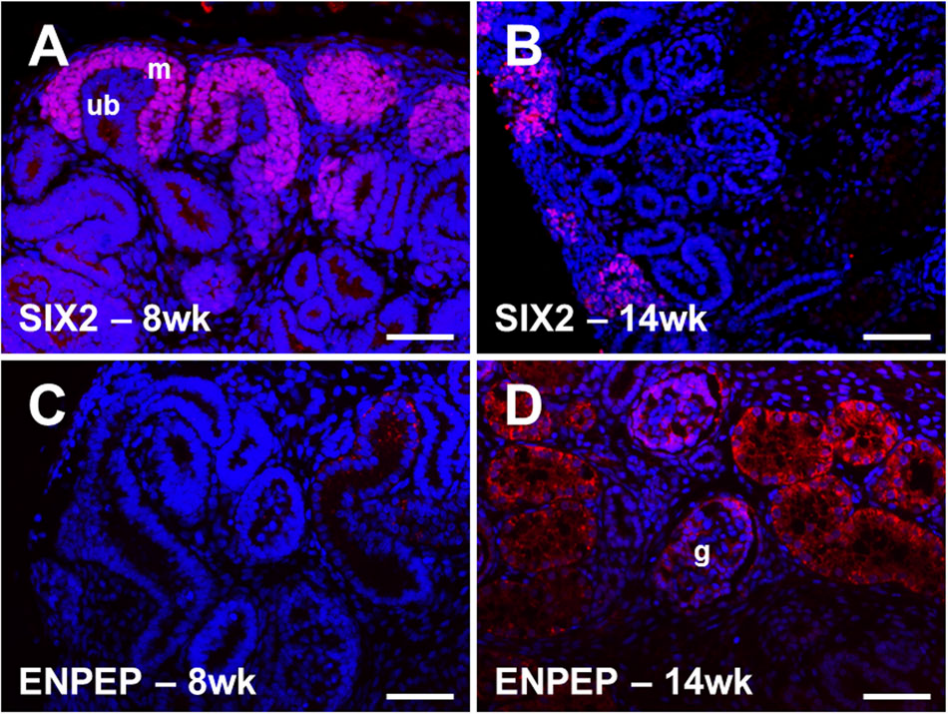
Expression of SIX2 and ENPEP in developing human metanephroi. **a** At 8 weeks of age, SIX2 localised to the mesenchyme (m) surrounding the ureteric bud (ub). **b** SIX2 expression was diminished at 14 weeks of gestation and restricted to mesenchyme at the periphery of the metanephros. **c** ENPEP was sparsely expressed in 8 week old metanephroi. **d** Expression was prominent in tubular epithelia at 14 weeks of gestation, and also detectable at a lower intensity within the glomerulus (g). Bar is 100 µm in all panels

Using sections of human foetal kidneys (gestational ages 8 and 14 weeks) we examined the localisation of SIX2 and ENPEP, two proteins whose expression was altered by lithium induced MET conversion. Similar to the expression patterns seen in the early developing mouse kidney^28^ SIX2 was localised to the condensed mesenchyme surrounding the ureteric bud at 8 weeks of age (Fig. 4a). Previous work from our laboratory^29^ also detected SIX2 protein in the mesenchyme of 9 and 12 week old foetal human kidneys. However, by 14 weeks of age, SIX2 expression was markedly diminished compared to earlier gestation, and restricted to mesenchyme cells in the periphery of the metanephros (Fig. 4b), a pattern which has also been observed by others in older foetal human kidneys^30^. ENPEP has not previously been described in early human nephrogenesis and we found only sparse expression with occasional epithelia positive for the protein at 8 weeks (Fig. 4c). ENPEP expression was more widespread at 14 weeks of gestation with prominent localisation in tubular epithelia and cells within the glomerulus (Fig. 4d); this is similar to that described for developing mouse kidneys^31^.

### Assessment of downstream targets following addition of lithium chloride

**Fig. 5:**
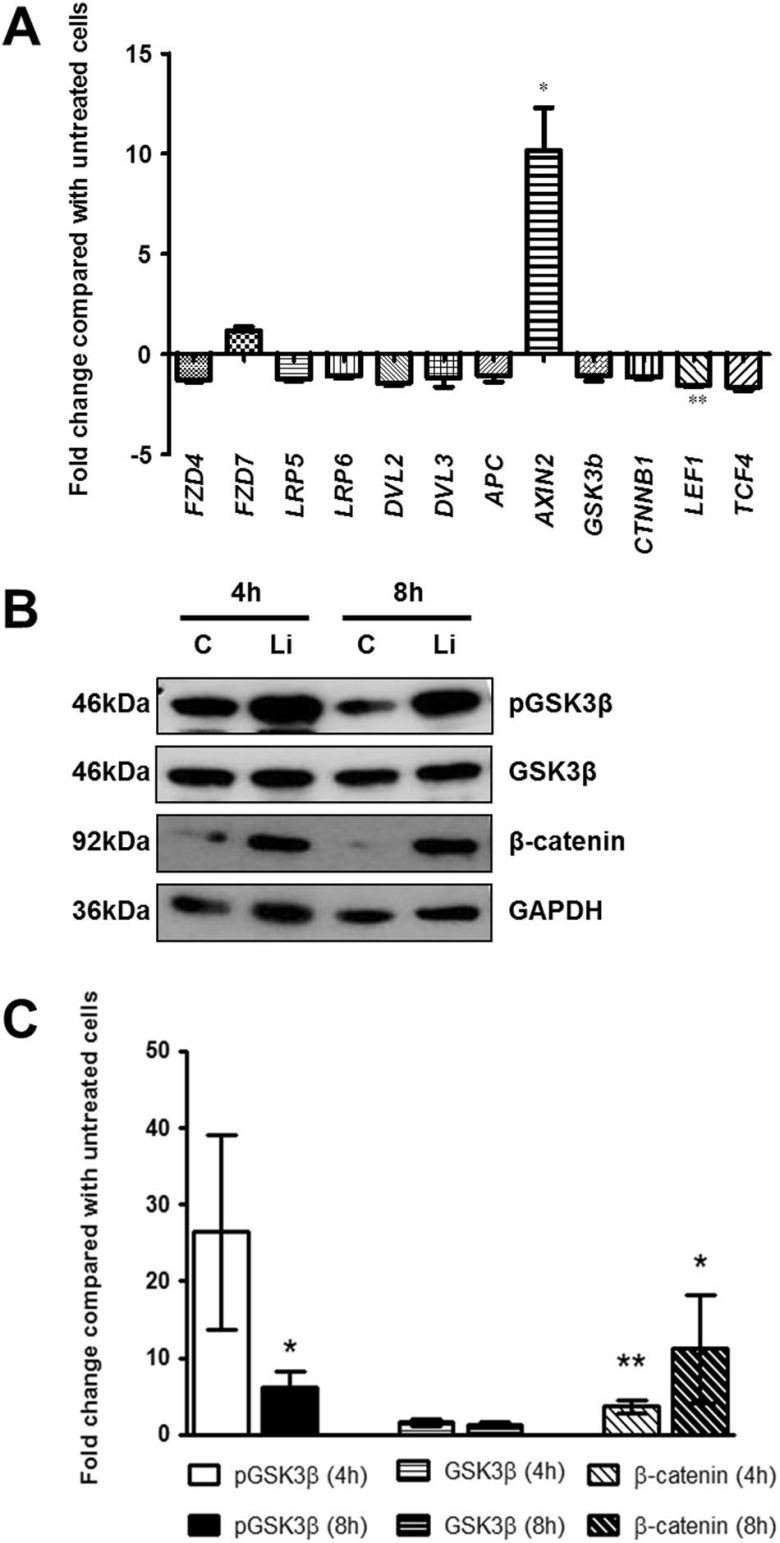
Effect of lithium chloride exposure on early Wnt signalling in foetal kidney mesenchymal cells. **a** qRT-PCR to assess the effect of 4 h of lithium chloride exposure on the mRNA levels of frizzled-4 (*FZD4)*, *FZD7*, low-density lipoprotein receptor-related protein 5 (*LRP5)*, *LRP6*, dishevelled 2 (*DVL2)*, *DVL3*, adenomatous polyposis coli (*APC)*, *AXIN2*, glycogen synthase kinase-3β (*GSK3B)*, β-catenin (*CTNNB1)*, transcription factor 4 (*TCF4)* and lymphoid enhancer binding factor 1 (*LEF1)* in foetal kidney mesenchymal cells. Data is expressed as mean ± SEM of the fold-change in mRNA expression in the lithium chloride treated cells relative to the mean intensity in the three independent untreated metanephric cell lines which were given a standardised value of 1. All measurements were performed in duplicate for three replicates of each cell line. *p < 0.05 and **p < 0.01 between lithium-treated and control-treated cells. **b** Assessment of total and phosphorylated (p) levels of GSK3β and total β-catenin in foetal mesenchymal cells with (Li)/without (**c**) lithium chloride exposure for 4 or 8 h exposure. Glyceraldehyde 3-phosphate dehydrogenase (GAPDH) was used as a house-keeping protein. Images are representative of experiments performed on three independent cell lines. Full length blots are shown in Supplementary Fig. 2. **c** Densitometry analysis of total and phosphorylated levels of GSK3β and total β-catenin normalised to GAPDH. Data is expressed as mean ± SEM of the fold-change in protein expression in the lithium chloride treated cells relative to the mean intensity in the three independent untreated metanephric cell lines which were given a standardised value of 1. Data was log-transformed to normalise distribution prior to statistical analysis. *p < 0.05 and **p < 0.01 between lithium-treated and control-treated cells

The prior studies in rodents demonstrated that addition of exogenous lithium led to activation of Wnt signalling^10^. Therefore, we analysed this pathway in the mesenchymal cell lines treated with 20 mM lithium chloride. We focused on changes 4 h after lithium chloride to identify early molecular alterations prior to phenotypic changes in the cells. Firstly, we examined the mRNA levels of the Wnt receptors, frizzled-4 (*FZD4)* and *FZD7*, their co-receptors low-density lipoprotein receptor-related protein 5 (*LPR5)* and *LRP6*, and downstream targets dishevelled 2 (*DVL2)* and *DVL3*^32^. There was no difference in the expression of these genes between control and lithium chloride treated cells (Fig. 5a). We then assessed the components of the destruction complex (adenomatous polyposis coli (*APC)*, *AXIN2*, and *GSK3B*)) which is involved in degradation of β-catenin unless Wnt signalling is activated^32^. We found that addition of 20 mM lithium chloride for 4 h led to a significant 10.2 ± 2.1-fold increase in *AXIN2* transcript levels compared with control foetal mesenchymal cells (*p* < 0.05), but *APC* and *GSK3B* were not altered (Fig. 5a). Four hours of exposure to 20 mM lithium chloride did not alter mRNA levels of β-catenin (*CTNNB1)* compared with control mesenchymal cells. There was also no change in the Wnt target gene transcription factor 4 (*TCF4)*^33^, but we found a small but significant reduction in mRNA levels of lymphoid enhancer binding factor 1 (*LEF1)* (1.6 ± 0.2-fold decrease, *p* < 0.01) in lithium exposed mesenchymal cells compared with controls (Fig. 5a).

To further examine Wnt signalling, we prepared protein extracts at 4 h following lithium chloride exposure and a later time-point of 8 h to assess the phosphorylation of GSK3β. Each of the three lines showed an increase in phosphorylation of GSK3β in lithium-treated vs. control foetal mesenchymal cells at four hours (Fig. 5b, Supplementary Fig. 2). However, there were also marked differences between the lines in basal levels of phosphorylated GSK3β. This variability may have contributed to the non-significant *p*-value of 0.08 when data was aggregated from the independent lines (Fig. 5c). GSK3β phosphorylation was significantly increased after 8 h of stimulation with a 6.1 ± 2.1-fold elevation in lithium exposed cells (*p* < 0.05). In contrast, there were no changes in the total amount of GSK3β protein at either time-point (Fig. 5b–c). We also examined the protein levels of total β-catenin. Exposure of foetal mesenchymal cells to lithium chloride led to a consistent increase (4 h: 3.7 ± 0.8-fold, *p* < 0.01; 8 h: 11.2 ± 7.1-fold, *p* < 0.05) in total β-catenin in all of the three cell lines examined (Fig. 5b–c).

## Discussion

During normal nephrogenesis, mesenchymal cells in the foetal kidney give rise to all of the nephron epithelia proximal to the collecting ducts, along with the renal stroma and components of the vasculature^3^. Moreover, cells derived from this lineage also have the ability to de-differentiate, repopulate tubules and contribute to repair, at least in rodent models of kidney injury^3^. These mesenchymal-derived cells come from several different locations including the renal papilla^34^, and Bowman’s capsule^35^, as well as classical epithelia in the proximal tubule^36^.

In this study, we firstly assessed the capacity of foetal mesenchymal cells to differentiate into different lineages. These studies were performed in triplicate for three different cell lines from 10 week old gestation kidneys to ensure reproducibility. We demonstrated phenotypic and molecular differentiation towards an osteogenic lineage, but not to adipogenic or endothelial cells. The mesenchyme contains vascular precursors in vivo^37^, therefore it was surprising that these cells did not endothelialise. However, renal vessel formation is by a combination of vasculogenesis and angiogenesis^37^, so it may be that any vascular precursors need additional stimuli from ingrowing cells that are lacking in this in vitro differentiation assay. Our findings confirm that our three independent foetal mesenchymal cell lines are all able to differentiate towards an osteogenic, but not adipogenic or endothelial lineages i.e., they have some extra-renal differentiation potential, but they are not pluripotent. In future experiments, it would be interesting to assess whether any of the initial cells express endothelial makers such as platelet endothelial cell adhesion molecule (CD31) or vascular endothelial growth factor receptor 2 (VEGFR2), which were then lost during culture. It would also be informative to sort cells for purported progenitor markers such as NCAM-1^38^, CD24^23^ and CD133^21^ and assess their differentiation potential. Further studies might also test additional differentiation protocols, such as chrondrogenic^39^ and neuronal^34^ potential.

Leukaemia Inhibitory factor (LIF) promotes MET in foetal rat renal mesenchyme^40^, but we previously demonstrated that this does not work with human cells^15^. Going back to evidence from animal experiments, lithium exposure is also able to promote epithelialisation, perhaps more effectively than LIF since they work in mice, as well as rats. They also act primarily through two different molecular pathways with LIF activating the Jak-Stat pathway^41^, whereas lithium affects Wnt signalling^10^. In our study, we also found that human foetal renal mesenchymal cells could undergo MET following lithium stimulation, showing parallels with previous observations in rodents^10, 11^. This appears to be specific, rather than an osmotic effect because addition of potassium chloride did not lead to MET. This is the first time that MET has been reported with human foetal renal cells exposed to lithium, and it identifies a common mechanism conserved between species. Moreover, there was not just generic epithelial change but specific proximal tubule differentiation with cells expressing the brush border enzyme ENPEP. This could be further examined via functional assays such as anion or cation uptake^42, 43^.

We found that just 24 h of lithium treatment was sufficient to promote MET in longer term culture. This suggests that early Wnt stimulation by lithium chloride, rather than sustained signalling, is sufficient to drive epithelisation. Therefore, we examined early molecular changes after 4 and 8 h of exposure to 20 mM lithium chloride. Since lithium acts by inhibiting GSK3β^44^, we did not expect upstream components of the Wnt pathway to be affected, and this was confirmed by a lack of changes in the mRNA levels of Wnt receptors (*FZD4, FZD7)*, co-receptors (*LPR5, LRP6)* and their immediate targets (*DVL2, DVL3*). Instead we found a strong upregulation in the mRNA levels of *AXIN2*, a component of the destruction complex which negatively regulates Wnt signalling by promoting the phosphorylation and degradation of β-catenin. *AXIN2* is also a target gene for nuclear β-catenin^45^, hence it may in turn be downregulated when Wnt signalling is activated via a negative feedback loop that might limit the duration or intensity of Wnt stimulation following lithium exposure. We also found that lithium chloride did not change *TCF4* mRNA levels compared with control mesenchymal cells, but *LEF1* showed a significant decrease. Both *TCF4* and *LEF1* are Wnt target genes but differential expression changes have been demonstrated in other models: as an example, in melanoma studies, LEF1 is prominent in differentiated/proliferative cells whilst dedifferentiated/invasive cells express TCF4^46^.

Lithium chloride treatment did not alter either *GSK3B* or *CTNNB1* mRNA, but protein phosphorylation is often considered more important than mRNA levels in this signalling pathway; hence, we examined phosphorylation by Western blotting. As expected, lithium chloride increased the phosphorylation of GSK3β after 4 h in each cell line and this was significant at 8 h compared with control foetal mesenchymal cells, whilst total GSK3β protein was unchanged. Furthermore, lithium exposure also significantly increased the total protein levels of β-catenin. These results were consistent across all three cell lines and provide further evidence that lithium is working via the Wnt pathway.

A future refinement of these differentiation studies could be sequential treatment with the GSK-3β inhibitor CHIR99021 followed by FGF9, a protocol which successfully leads to nephron formation from induced pluripotent stem cells^47^. Alternatively, since three dimensional structures may be critical as suggested by neo-organoid nephrogenesis experiments^48, 49^, growing aggregates of cells with or without lithium may be informative.

In conclusion, we have demonstrated that MET, the essential first step in nephron generation, can be promoted by chemical stimulation of human foetal kidney mesenchymal cells with lithium. This demonstrates a common Wnt-dependent mechanism between humans and rodents, unlike other inducers such as LIF which do not generate the same results across species^15^.

## Materials and methods

All reagents were obtained from Sigma Chemical Company (Poole, UK) unless otherwise stated.

### Ethics

The human embryonic and foetal material was provided by the Joint MRC/Wellcome Trust grant #099175/Z/12/Z Human Developmental Biology Resource (http://hdbr.org). The HDBR obtains written consent from the donors and has ethics approval (REC reference: 08/H0712/34 + 5) to collect, store and distribute human embryonic and foetal material between 4 and 20 weeks post conception. All experimental protocols were approved by the Institute’s Ethical Committee (Reference 010/H0713/6) and were performed in accordance with institutional ethical and regulatory guidelines.

### Cell culture

Human metanephric cell lines (*n* = *3*) were generated as previously described^15, 16^ from 10 week gestation foetuses obtained from the ethically approved MRC/Wellcome-funded Human Developmental Biology Resource (http://www.hdbr.org)^50^. Briefly, the kidneys were diced and placed in normal basal growth media, comprised of Dulbecco’s Modified Eagle Medium (DMEM)/Hams F12 (1:1) mix (Thermo Fisher Scientific, Waltham, MA, USA) supplemented with 5% foetal calf serum (FCS; Thermo Fisher Scientific), prostaglandin E1 (100 ng/ml), dexamethasone (40 ng/ml), 3,3′,5-Triiodo-L-thyronine sodium salt (4 pg/ml), holo transferrin (20 ng/ml), insulin/transferring/selenium liquid media supplement (100×), penicillin G (1000 U/L) streptomycin (1 mg/ml) and amphotericin (25 mg/ml). Cells were cultured at 37 °C in a 5% CO_2_ incubator, media was changed twice weekly and confluent cells passaged 1:3 or 1:5.

### Differentiation potential of human foetal kidney mesenchymal cells

To assess osteogenic differentiation potential, cells (*n* = 3 independent lines, each performed in triplicate) were seeded at a density of 5 × 10^4^ in a 24-well plate, treated with either normal growth media (control group) or StemMACS OsteoDiff Media (Miltenyi Biotec, Bisley, UK) for 12 days and fixed for Alizarin red staining. In addition, cells were harvested for preparation of RNA and subsequent detection of genes which promote osteogenic differentiation (*RUNX2*, *BMP2*, *SP7* and *BLGLAP*)^18^. Adipogenic differentiation potential was assessed by seeding 5 × 10^4^ cells/well in a 24-well plate and culturing them for 21 days in StemMACS AdipoDiff Media (Miltenyi Biotec Ltd); media was changed every three days. Oil red staining was then performed to detect lipid droplets. To investigate endothelial differentiation potential, cells (1 × 10^5^/well) were seeded on matrigel coated 6-well plates and cultured for 7 days in EGM2 with supplements (Lonza, Slough, UK). To assess epithelial differentiation potential, cells (1 × 10^5^/well) were seeded on 6-well plates and cultured for up to 7 days in normal growth media supplemented with lithium chloride (1 mM and 20 mM); some cells were also exposed to potassium chloride (20 mM) to check if alterations in osmotic environment leads to differentiation. Additional experiments were performed treating the cells in normal media with/without 20 mM of LiCl for 24 h and subsequently exposing the cells to normal growth media for the subsequent six days. At the end of the experimental period, cells were either harvested for RNA or protein. In some experiments, media was also collected to assess the secretion of HGF.

### Alizarin red staining

To detect calcium deposits, cells were fixed in 4% paraformaldehyde (PFA) for 15 min, washed twice with phosphate buffered saline (PBS) and exposed to a 2% Alizarin Red solution for 10 min with shaking. Cells were then washed again with PBS to remove the excess stain, and images captured.

### Oil red staining

To detect lipid droplets cells were fixed in 4% PFA for 15 min, rinsed with sterile water, incubated with 60% isopropanol solution for 5 min and exposed to Oil Red O solution (3 parts Oil Red O with 2 parts water) for 15 min. Cells were washed again with sterile water to remove the excess stain, and images captured.

### RT-PCR and qPCR

RNA was extracted with Tri-Reagent and 1 µg used to prepare cDNA. In foetal kidney mesenchymal cells treated with either normal growth media or StemMACS OsteoDiff Media) for 12 days RT-PCR was performed for *BLGLAP*, *BMP2, RUNX2* and *SP7* using iTaq DNA polymerase (Bio-Rad Laboratories, Hemel Hempstead, UK) with *HPRT* as a house-keeping gene^15^. Following lithium chloride treatment, quantitative real-time RT-PCR was performed for *APC*, *AXIN2*, *CD133*, *CD24*, *CDH1*, *CTNNB1*, *DVL2*, *DVL3*, *ENPEP*, *EYA1*, *FGF8*, *FZD4*, *FZD7*, *GSK3B*, *HGF*, *LEF1*, *NPHS1*, *NPHS2*, *SIX2*, *TCF1*, *WNT4* and *WNT9B* on a CFX96 Real-Time PCR System (Bio-Rad Laboratories) using SsoAdvanced Supermix (Bio-Rad Laboratories) with β-actin used as a house-keeping gene. For each of the three cell lines, fold-changes in gene expression in the treated cells were determined by the 2^−ΔΔ*C*T^ method and expressed relative to the mean intensity in the cells grown in normal basal media, which was given a standardised value of 1. All measurements were performed in duplicate for three replicates for each cell line. The presented data shows the mean fold-change in gene expression measured in three independent cell lines. Primer details available on request.

### Western blot

Cells were lysed in RIPA buffer and Western blotting performed using 15 µg of protein as previously described^8^. Following electrophoresis and transfer by electro-blotting, membranes were blocked in 5% non-fat milk for one hour prior to incubation with either: rabbit anti-human phosphorylated GSK3β (9323, New England Biolabs, Hitchin, UK), rabbit anti-human total GSK3β (9315, New England Biolabs) or rabbit anti-human total β-catenin (9562, New England Biolabs) antibodies overnight at 4 °C. Appropriate horseradish peroxidase antibodies were then used and bands detected by chemiluminescence (GE Healthcare Life Sciences, Little Chalfont, UK). Blots were stripped and reprobed with mouse anti-rabbit GAPDH (MAB374, Millipore) as a housekeeping protein. Densitometry was performed using ImageJ analysis (http://rsbweb.nih.gov.ij/). For each of the three cell lines, fold-changes in protein expression in the treated cells was expressed relative to the intensity of the cells grown in normal basal media, which was given a standardised value of 1. The presented data shows the mean fold-change in protein expression measured in three independent cell lines.

### ELISA

Media from the human foetal kidney mesenchymal cells (*n* = *3* independent lines) was collected after 7 days of exposure to normal growth media with or without 20 mM lithium chloride. The media was centrifuged to remove any cell debris and HGF secretion assessed by ELISA (R&D Systems Europe, Abingdon, UK) according to the manufacturer’s instructions. Optical density was determined at 450 nm and the HGF protein content calculated using a standard curve derived from known concentrations of recombinant protein. All readings were then standardised to the protein content of the cells as assessed by the bicinchoninic acid protein assay (Thermo Fisher Scientific). For each cell line, the experiment was repeated three times and all assays performed in duplicate; HGF levels were expressed relative to those detected in the cells grown in normal basal growth media, which were standardised to a value of 1.

### Immunohistochemistry

Normal human metanephroi (8 and 14 weeks gestation) were fixed in 4% paraformaldehyde, embedded in paraffin and sectioned at 5 µm. Sections were then dewaxed, rehydrated and antigens unmasked by microwave treatment with 10 mM sodium citrate (dihydrate, pH 6.0). Non-specific binding was blocked by incubation with 10% FCS in PBS containing 0.5% bovine serum albumin (BSA) and 0.1% Tween-20 for 60 min prior to overnight incubation at 4 °C with either goat anti-human ENPEP (PA5-18393, Thermo Fisher Scientific) or rabbit anti-mouse SIX2 (ab68908, Abcam, Cambridge, UK) antibodies. As negative controls, primary antibodies were omitted. After washing in PBS containing 0.1% Tween-20, sections were incubated with appropriate AlexaFluor594 secondary antibodies and nuclei stained by Hoechst dye.

### Immunocytochemistry

5 × 10^3^ cells/well were plated out in 200 µl of normal growth medium with/without 20 mM lithium chloride on four-well Lab-Tek chamber slides (Thermo Fisher Scientific) coated with fibronectin and incubated at 37 °C for 7 days. Immunocytochemistry was performed as described^51^. Cells were fixed with 4% PFA and non-specific binding sites were blocked by incubation with 10% FCS, 0.2% BSA and 0.1% Tween-20 in PBS for 30 min. Cells were then incubated overnight at 4 °C with a mouse anti-human antibody against pan cytokeratin (Abcam) followed by appropriate AlexaFluor594 secondary antibodies and Hoechst dye to stain nuclei.

### Statistics

Data are presented as mean ± SEM and were analysed using GraphPad Prism (GraphPad Software, La Jolla, CA). Differences between control and treated cells were analysed using a *t*-test. For Western blot analysis, the data was not normally distributed and the results were log transformed prior to statistical analysis. Statistical significance was accepted at *p* < *0.05*.

## Electronic supplementary material

Supplementary Data
