# Supplementary material for: Lithium induces mesenchymal-epithelial differentiation during human kidney development by activation of the Wnt signalling system
Source: Cell Death Discov. 2018 Feb 7;4:13. doi: 10.1038/s41420-017-0021-6 (PMC5841285; doi:10.1038/s41420-017-0021-6)

**Supplementary Data**

**Lithium induces mesenchymal-epithelial differentiation during human kidney development by activation of the Wnt signalling system**

Karen L. Price, Maria Kolatsi-Joannou, Chiara Mari, David A. Long, Paul J.D. Winyard.

Developmental Biology and Cancer Programme, UCL Great Ormond Street Institute of Child Health, London, UK


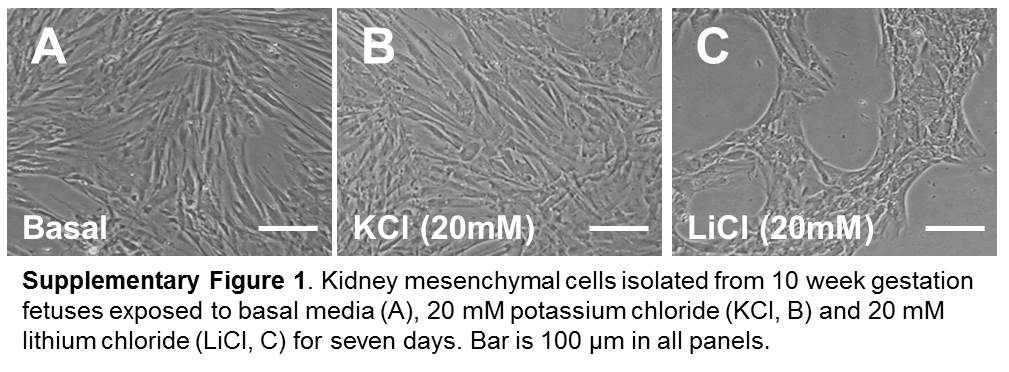


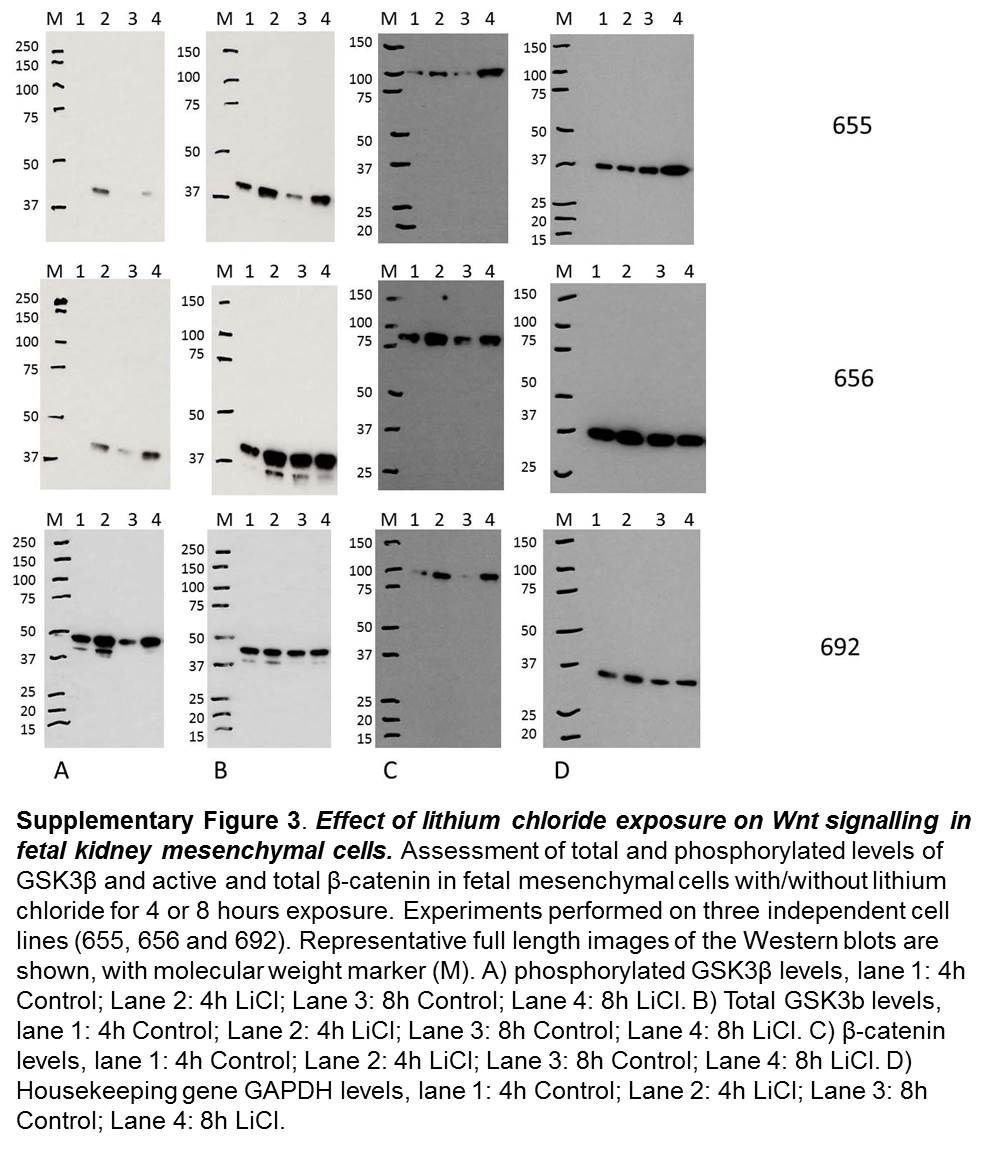

Supplement: Supplementary file 1 — Supplementary Data [file 41420_2017_21_MOESM1_ESM.docx]
